# Plasma proteomic profiling of young and old mice reveals cadherin-13 prevents age-related bone loss

**DOI:** 10.18632/aging.103184

**Published:** 2020-05-12

**Authors:** Yong Ryoul Yang, Mohammad Humayun Kabir, Jin Hee Park, Jae-Il Park, Jae Sook Kang, Shinyeong Ju, Yeo Jin Shin, Seung Min Lee, Jaemin Lee, Seokho Kim, Kwang-Pyo Lee, Soo Young Lee, Cheolju Lee, Ki-Sun Kwon

**Affiliations:** 1 Aging Research Center, Korea Research Institute of Bioscience and Biotechnology, Daejeon, Republic of Korea; 2 Center for Theragnosis, Korea Institute of Science and Technology, Seoul, Republic of Korea; 3 The Research Center for Cellular Homeostasis, Department of Life Science, Ewha Womans University, Seoul, Republic of Korea; 4 Korea Basic Science Institute, Gwangju Center at Chonnam National University, Gwangju, Republic of Korea; 5 Department of Functional Genomics, KRIBB School of Bioscience, Korea University of Science and Technology (UST), Daejeon, Republic of Korea; 6 Department of Life Science and Research Institute for Natural Sciences, Hanyang University, Seoul, Republic of Korea; 7 Department of Medicinal Biotechnology, College of Health Sciences, Dong-A University, Busan, Republic of Korea; 8 KHU-KIST Department of Converging Science and Technology, Kyung Hee University, Seoul, Republic of Korea; 9 Division of Bio-Medical Science and Technology, KIST School, Korea University of Science and Technology, Seoul, Republic of Korea; 10 Present address: Incepta Vaccine Limited, Dhamrai, Bangladesh

**Keywords:** aging, bone, plasma proteins, proteomics, osteoclast differentiation

## Abstract

The blood exhibits a dynamic flux of proteins that are secreted by the tissues and cells of the body. To identify novel aging-related circulating proteins, we compared the plasma proteomic profiles of young and old mice using tandem mass spectrometry. The expression of 134 proteins differed between young and old mice. We selected seven proteins that were expressed at higher levels in young mice, and confirmed their plasma expression in immunoassays. The plasma levels of anthrax toxin receptor 2 (ANTXR2), cadherin-13 (CDH-13), scavenger receptor cysteine-rich type 1 protein M130 (CD163), cartilage oligomeric matrix protein (COMP), Dickkopf-related protein 3 (DKK3), periostin, and secretogranin-1 were all confirmed to decrease with age. We then investigated whether any of the secreted proteins influenced bone metabolism and found that CDH-13 inhibited osteoclast differentiation. CDH 13 treatment suppressed the receptor activator of NF-κB ligand (RANKL) signaling pathway in bone marrow-derived macrophages, and intraperitoneal administration of CDH-13 delayed age-related bone loss in the femurs of aged mice. These findings suggest that low plasma CDH-13 expression in aged mice promotes aging-associated osteopenia by facilitating excessive osteoclast formation. Thus, CDH-13 could have therapeutic potential as a protein drug for the prevention of osteopenia.

## INTRODUCTION

Aging is a time-dependent functional decline that is characterized by the progressive loss of physiological integrity, leading to organ dysfunction and increased vulnerability to age-related diseases [1]. With age, the plasma proteins secreted by tissues change dramatically [2–4]. Since plasma proteins reflect human physiological or pathological states, they are a potential goldmine of candidate biomarkers for age-related changes [5]. The study of such markers could clarify the physiological processes of aging, which in turn could provide insights into potential therapeutic targets and strategies to alleviate the effects of aging [6, 7].

Numerous studies have demonstrated that young plasma can reverse aging phenotypes and rejuvenate the aged brain, muscles, bones, liver and heart [8–11]. Various approaches have been used to identify circulating proteins that are differentially expressed and/or involved in organ dysfunction during aging. CCL11 (a chemokine also known as eotaxin) and β2-microglobulin are upregulated in aged humans and mice, and these increases are speculated to impair cognitive function [12, 13]. Conversely, thrombospondin-4 (THBS4) and SPARC-like protein 1 (SPARCL1) are enriched in the plasma of young mice, and act directly on neurons as synaptogenic factors [14]. The plasma levels of oxytocin and apelin decrease with age, which is notable because oxytocin deficiency leads to premature sarcopenia in mice [15]. The injection of aged mice with recombinant apelin was reported to reverse age-related losses of muscle mass and strength [16]. These findings suggest that specific factors in young or old plasma can contribute to the aging process and/or age-related diseases.

With advancing age, osteoclast-induced bone resorption outpaces osteoblast-induced bone deposition, leading to a gradual loss of bone mass. The use of therapeutic agents that inhibit osteoclast activity and differentiation has been proposed as a strategy to prevent osteoporosis and other bone-related diseases. Osteoclast differentiation is induced by macrophage-colony stimulating factor (M-CSF), receptor activator of nuclear factor (NF)-κB ligand (RANKL) and osteoprotegerin [17]. These cytokines are involved in signaling pathways that balance the activities of osteoblasts and osteoclasts to maintain bone mass homeostasis. The monoclonal antibody denosumab is the only RANKL inhibitor currently approved by the US Food and Drug Administration, and has been reported to reduce bone turnover and increase bone mineral density (BMD) [18].

Cadherin-13 (CDH-13, also known as T-cadherin or H-cadherin) is an atypical member of the cadherin superfamily. It lacks the typical transmembrane and cytoplasmic domains of this superfamily, and is linked to the plasma membrane via a glycosylphosphatidylinositol moiety [19, 20]. CDH-13 is abundant in developing and adult brains [21], and has been associated with various psychiatric disorders [22–24]. In addition, CDH-13 influences vascular function, and has been associated with vascular disorders such as atherosclerosis and hypertension [25–27]. Although CDH-13 is known to regulate cell-cell interactions in the plasma membrane, it also circulates in the blood. Elevated plasma CDH-13 levels have been associated with early atherosclerosis development [26], but reduced plasma CDH-13 levels have been associated with a greater severity of coronary artery disease and a higher risk of acute coronary syndrome [28]. Thus, the function of circulating CDH-13 is not fully understood.

Given that plasma proteins include aging-related factors, we hypothesized that aging would dynamically alter the plasma levels of proteins involved in age-related bone loss. We used a proteomic approach to identify plasma proteins that were differentially expressed between young and old mice, and investigated their effects on osteoclasts and osteoblasts. We focused on CDH-13, examining its impact on osteoclast differentiation and bone resorption. Finally, we tested whether intraperitoneal administration of CDH-13 could prevent age-related bone loss.

## RESULTS

### Plasma proteome profiling of young and old mice

**Figure 1:**
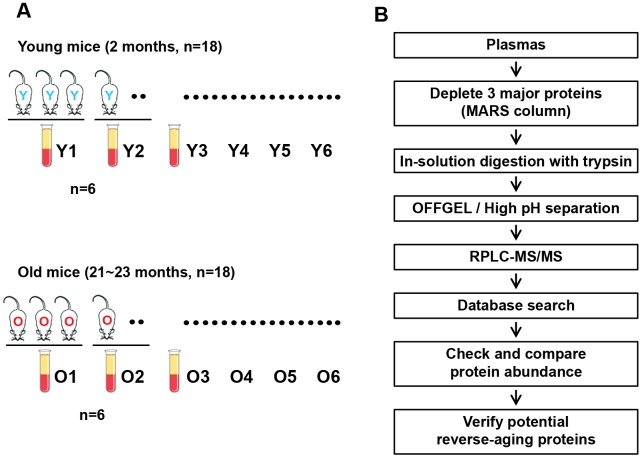
**Overall workflow of the proteomic profiling of young versus old mouse plasma.** (**A**) Plasma samples were collected from 18 young (2-month-old) and 18 aged (21- to 23-month-old) C57BL/6J mice, and plasma samples from trios of mice were combined to generate six pooled sets per group. (**B**) Flowchart of the proteomic analysis of mouse plasma and the validation of reverse-aging candidate proteins.

The overall workflow of this study is outlined in Figure 1. We collected plasma samples from 18 young (2-month-old) and 18 aged (21- to 23-month-old) C57BL/6J mice, and pooled the samples of every three mice (six pools per group) for subsequent experiments (Figure 1A). The most abundant mouse plasma proteins (albumin, IgG and transferrin) were depleted by immunoaffinity chromatography. The remaining plasma proteins (300 μg each) were digested with trypsin and separated into 12 fractions using isoelectric-point (pI)-based OFFGEL separation and high-pH reversed-phase liquid chromatography (RPLC). Each fraction was further analyzed using nanoRPLC-tandem mass spectrometry (MS/MS). The mass spectra were searched against the SwissProt mouse protein database. Comparative protein quantitation was performed with the spectral count values (Figure 1B).

### Protein identification in young and old mouse plasma

**Figure 2:**
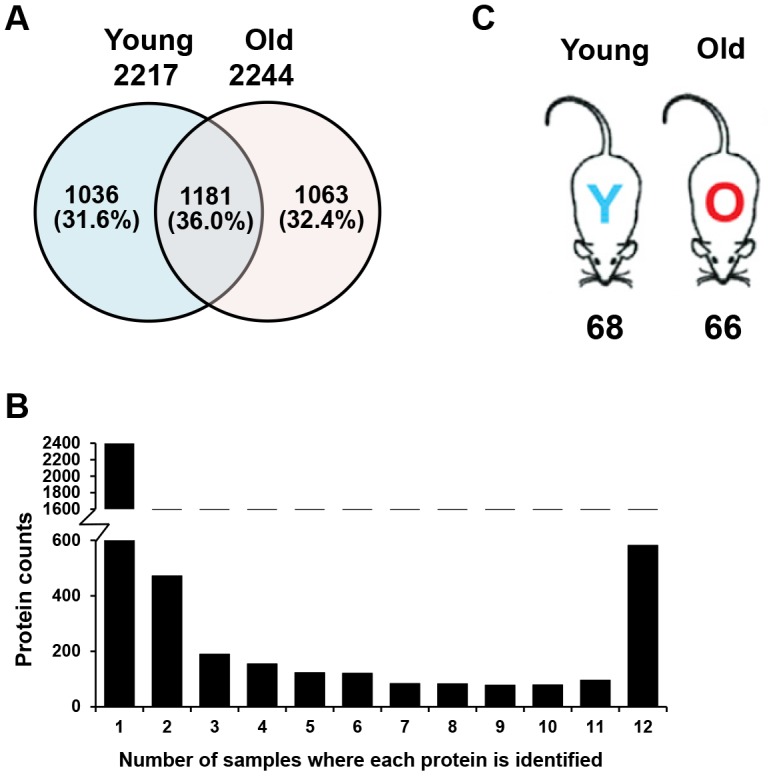
**Comparison of plasma proteins between young and old mice.** (**A**) A Venn diagram is shown for the identified proteins, which included 2217 proteins from young mouse plasma, 2244 proteins from old mouse plasma, and a total of 3280 plasma proteins. (**B**) The graph displays the number of samples in which each identified protein was found. Many of the proteins were identified in only a single sample, while nearly 600 proteins were found in all 12 samples. (**C**) Number of proteins expressed at different levels in young and old mice. Statistical analyses were performed with the G-test and SAM.

In total, 288 liquid chromatography (LC)-MS/MS analyses were performed: 24 (2 separation methods × 12 sub-fractions) for each of the six replicates in both young and old mouse plasma samples. We identified 2217 proteins in young mouse plasma, 2244 proteins in old mouse plasma, and a total of 3280 proteins (Figure 2A, Supplementary Table 1). Many proteins were only identified in a single sample (Figure 2B), indicating that numerous proteins are present at very low levels in plasma and are difficult to quantify due to their variability among mice [29, 30]. However, nearly 600 proteins were found in all 12 pooled plasma samples, and some of them varied in concentration between young and old mice. This indicates that the plasma proteome may change with age.

### Quantification of age-related protein abundances through spectral counts

The abundances of the proteins and peptide-spectrum matches identified in the six sets of young mouse plasma and old mouse plasma were compared based on their G-test and Significance Analysis of Microarrays (SAM) values. When G-values ≥ 3.841 and SAM values of d ≥ 1.96 were both applied as acceptance criteria, 134 abundant proteins were identified (Supplementary Table 1). Among them, 68 proteins were identified in young mouse plasma, while the remaining 66 proteins were identified in old mouse plasma (Figure 2C). It is well known that 22 high-abundance proteins, including albumin and immunoglobulin, make up 99% of plasma proteins [29]. Most of the proteins screened in our analysis were not members of this abundant protein group, and thus were probably present at relatively low levels. This demonstrates that lower-abundance proteins may be important contributors to the aging process.

### Age-related changes in candidate proteins in mouse plasma

**Figure 3:**
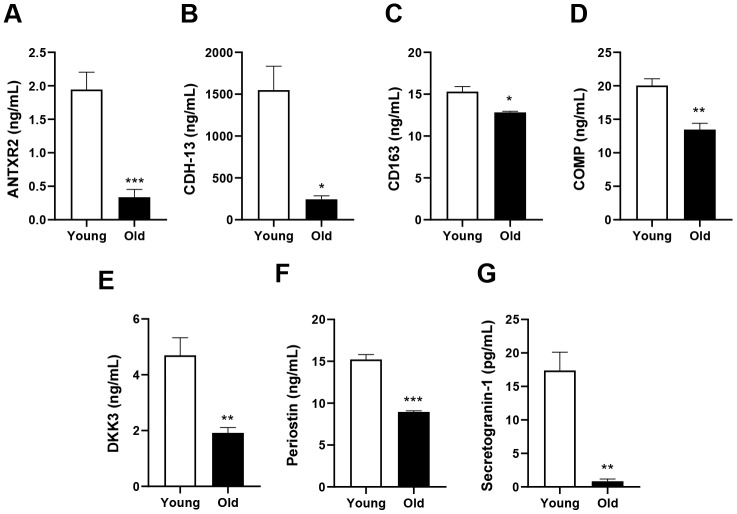
**Validation of the selected candidates in plasma from young and old mice**. Plasma concentrations of (**A**) ANTXR2, (**B**) CDH-13, (**C**) CD163, (**D**) COMP, (**E**) DKK3, (**F**) periostin and (**G**) secretogranin-1. Error bars represent ± SEM. *** *P* < 0.001, ** *P* < 0.01, * *P* < 0.05; NS, not significant.

From the plasma proteins identified above, we selected seven candidates that were downregulated in aged mice and had not been studied previously in aging-related research (Table 1). We prioritized proteins that contained signal peptides or were previously reported to be circulating proteins. The candidate proteins were anthrax toxin receptor 2 (ANTXR2), CDH-13, cartilage oligomeric matrix protein (COMP), scavenger receptor cysteine-rich type 1 protein M130 (CD163), Dickkopf-related protein 3 (DKK3), periostin and secretogranin-1. We used enzyme-linked immunosorbent assays to detect these candidates in the plasma of young and old mice, and found that all of them were expressed at lower levels in old mice than in young mice (Figure 3A–3G).

**Table 1:** Selected candidates for age-related changes in mouse plasma.

**Accession**	**Gene name**	**Description**	**pI-based separation**					**High pH separation**				
			**Total PSMs, Young**	**Total PSMs, Old**	**G-value**	**D value**	**Fold change (O/Y)**	**Total PSMs, Young**	**Total PSMs, Old**	**G-value**	**D value**	**Fold change (O/Y)**
Q6DFX2	Antxr2	Anthrax toxin receptor 2	48	20	10.2	4.5	0.417	11	6	N.S.	N.S.	0.545
Q9WTR5	Cdh13	Cadherin-13 (T-Cadherin)	34	30	N.S.	N.S.	0.882	36	16	6.1	3.1	0.444
Q9R0G6	Comp	Cartilage oligomeric matrix protein	78	38	12.2	3.7	0.487	44	24	4.3	2.6	0.545
Q9QUN9	Dkk3	Dickkopf-related protein 3	42	18	8.4	3.2	0.429	2	0	N.S.	N.S.	0.000
Q2VLH6	Cd163 M130	Scavenger receptor cysteine-rich type 1 protein M130	32	5	19.5	2.9	0.156	13	0	13.2	4.6	0.000
P47867	Scg3	Secretogranin-3	10	2	4.2	2.6	0.200	6	5	N.S.	N.S.	0.833
Q62009	Postn Osf2	Periostin	184	95	25.6	2.2	0.516	133	47	37.2	4.9	0.353

N.S., not significant or able to calculate

### CDH-13 inhibits osteoclast differentiation

**Figure 4:**
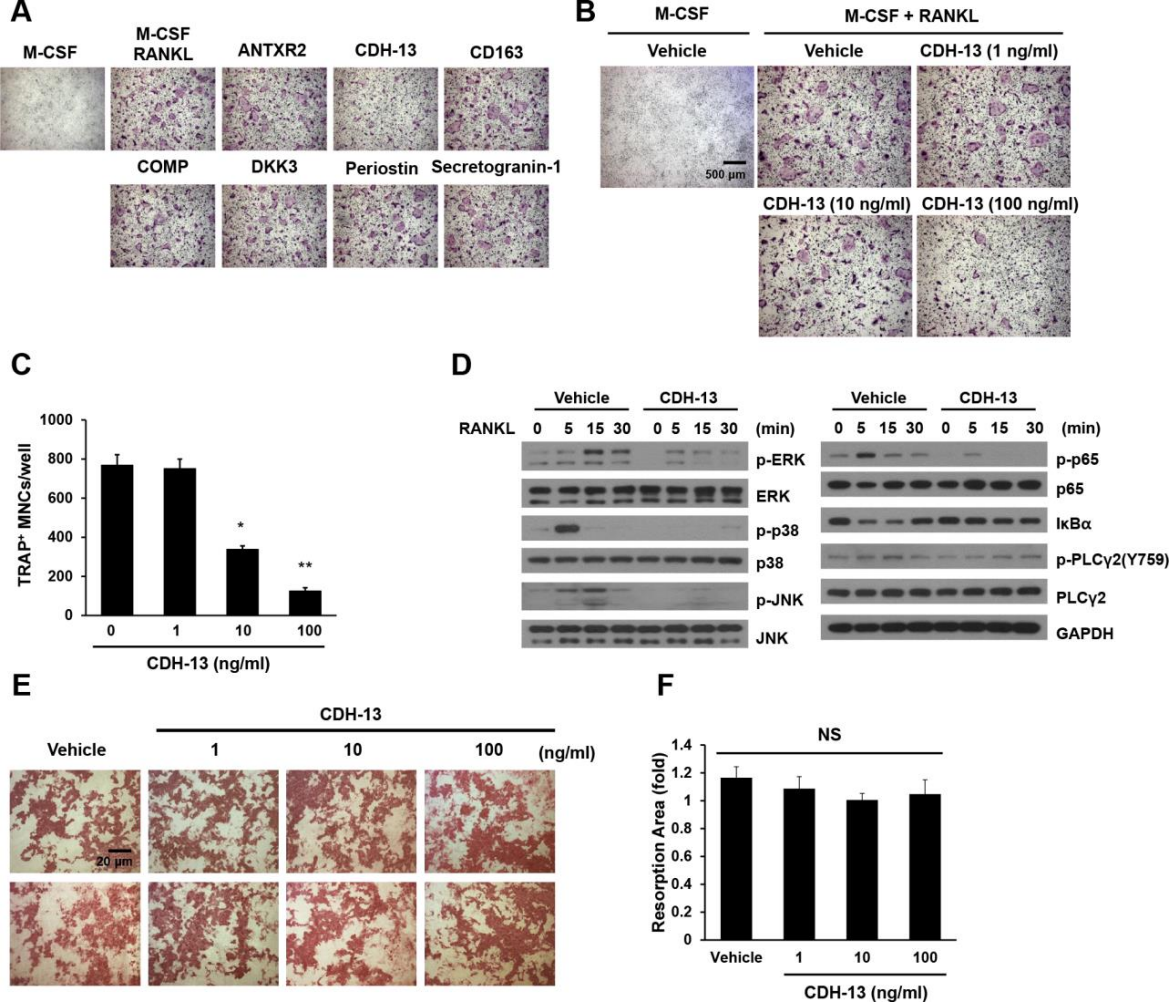
**Effects of CDH-13 on RANKL-induced osteoclast differentiation**. (**A**) BMMs were cultured for three days in the presence of M-CSF (30 ng/mL) and RANKL (100 ng/mL) with one of the candidate proteins (ANTXR2, CDH-13, CD163, COMP, DKK3, periostin or secretogranin-1; 100 ng/mL). Osteoclasts were stained with TRAP. (**B**) BMMs were incubated with various concentrations of CDH-13 (0, 1, 10 and 100 ng/mL). (**C**) TRAP-positive multinucleated cells with more than five nuclei were counted. (**D**) M-CSF-treated BMMs were pretreated with CDH-13 or the vehicle for 30 min. RANKL (100 ng/mL) was used to stimulate the cells at the indicated times, and immunoblotting was used to detect members of the RANKL/mitogen-activated protein kinase and NF-κB signaling pathways. (**E, F**) Differentiated osteoclasts were cultured in the presence of the vehicle or CDH-13 (1, 10 or 100 ng/mL) on dentin slices. Resorption pits were visualized with hematoxylin, and the resorption areas were measured. Error bars represent ± SEM. ** *P*< 0.01, * *P* < 0.05; NS, not significant.

We speculated that the candidate proteins might contribute to the aging process or the development of aging-associated diseases such as sarcopenia, osteopenia, cognitive decline, cardiovascular disease and so on. With increasing age, greater osteoclast formation or function can initially reduce the BMD. To test whether any of the identified proteins could inhibit osteoclast formation, we treated bone marrow-derived macrophages (BMMs) with each of the candidates during RANKL-induced osteoclast differentiation. Among the candidates, CDH-13, which was not toxic to the cells at any of the tested doses (Supplementary Figure 1), was found to inhibit osteoclast differentiation dose-dependently (Figure 4A–4C), while it did not inhibit osteoblast differentiation (Supplementary Figure 2A and 2B).

To assess the effects of CDH-13 on RANKL-associated signaling cascades, we examined the phosphorylation of signaling molecules in the mitogen-activated protein kinase and canonical NF-κB pathways. BMMs were pretreated with CDH-13 or PBS (the control) for 30 min, and then were stimulated with RANKL at the indicated time points. As shown in Figure 4D, RANKL rapidly induced the phosphorylation of extracellular signal-regulated kinase (ERK), p38, c-Jun N-terminal kinase (JNK), p65 and phospholipase C gamma 2 (PLCγ2), as well as the degradation of NF-κB inhibitor alpha (IκBα). CDH-13 pretreatment significantly inhibited the RANKL-induced phosphorylation/degradation of these signaling molecules (Figure 4D). These results suggest that CDH-13 blocks the initial activation of RANKL/RANK-induced signaling.

To determine whether CDH-13 treatment could also suppress osteoclast-induced bone resorption, we assessed pit formation in CDH-13-treated dentin slices (Figure 4E and 4F). However, CDH-13 treatment did not alter the area of bone resorbed by differentiated osteoclasts. These results indicate that CDH-13 inhibits osteoclast differentiation, but not osteoclast-induced bone resorption.

### CDH-13 administration delays bone loss in aged mice

**Figure 5:**
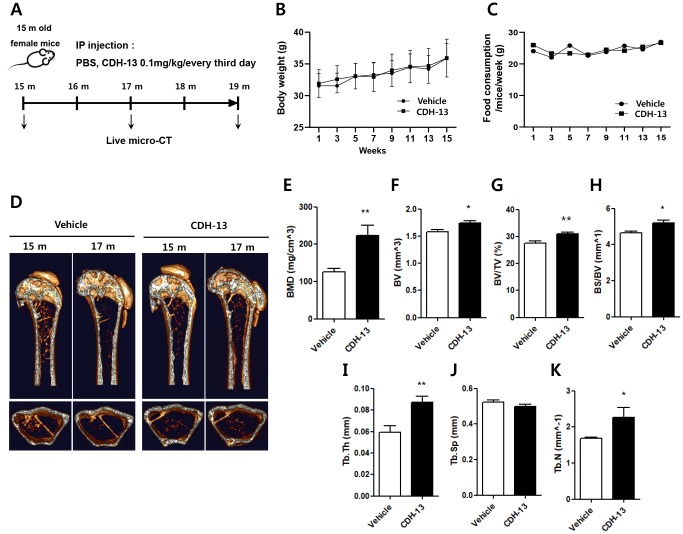
**Effects of CDH-13 on age-related bone loss in the distal femurs of old mice**. (**A**) Schematic representation of the experimental timeline for administering the vehicle (n = 5) or CDH-13 (n = 5) to 15-month-old mice. Changes in body weight (**B**) and dietary consumption (**C**) in the vehicle- and CDH-13-injected mice. (**D**) Representative micro-CT images of sagittal (*upper*) and transverse (*lower*) views of the distal femurs of vehicle- and CDH-13-injected mice. Histomorphometric analyses of (**E**) BMD, (**F**) bone volume, (**G**) trabecular bone volume over tissue volume, (**H**) bone surface area over bone volume, (**I**) trabecular thickness, (**J**) trabecular separation and (**K**) trabecular number in femurs from vehicle- and CDH-13-injected mice. Error bars represent ± SEM. ** *P* < 0.01, * *P* < 0.05.

To examine the possibility of using CDH-13 to treat age-related bone loss, we tested the effects of CDH-13 on bone homeostasis in old mice. Beginning at 15 months of age, female mice were intraperitoneally injected with CDH-13 or phosphate-buffered saline (the vehicle) for four months, as shown in the experimental timeline (Figure 5A). There were no differences in body weight or dietary consumption between the two groups of mice (Figure 5B and 5C). We used live-animal micro-computed tomography (micro-CT) for quantitative longitudinal analyses of bone loss (Figure 5D–5K). Over the 16 weeks following the primary injection, the BMD of the femur declined dramatically and progressively in the control mice. In the CDH-13-treated mice, this BMD decrease was attenuated, while the bone volume fraction and trabecular thickness were elevated (Figure 5E and 5F). CDH-13-treated mice showed higher trabecular bone volume (BV/TV) and bone specific surface (BS/BV) than the control mice (Figure 5G and 5H). The trabecular thickness and number were significantly higher in the CDH-13-treated mice than in the control mice (Figure 5I and 5K). The trabecular separation displayed a decreasing trend in the CDH-13-treated group, but the difference did not reach statistical significance (Figure 5J). These results demonstrate that CDH-13 administration can attenuate age-associated bone loss in aged mice, likely by blocking RANK-induced osteoclastogenesis.

## DISCUSSION

The characterization of aging-related proteins is an important step towards understanding the mechanisms of aging and age-related pathogenesis [4, 31]. Such analysis could also reveal diagnostic and prognostic biomarkers that could be used to reduce the burden of aging and to improve the health-span and productivity of senior citizens. Therefore, we assessed the plasma protein profiles of young and old mice, identified proteins that were downregulated in old mice, and verified their age-related characteristics. We adopted two different peptide separation/fractionation strategies for our proteomic analysis (OFFGEL separation and high-pH RPLC) in order to avoid method bias and to maximize the total number of identified proteins. We also applied two different statistical methods, which increased our confidence in the quantification results.

Although plasma proteins that change with aging have been documented in many ways, few studies have systematically explored and characterized the proteins that influence the aging process and age-related diseases. Several plasma proteome studies have used two-dimensional difference gel electrophoresis (2D-DIGE) approaches to identify age-related changes in plasma protein levels in humans and mice [32, 33], and Tanaka et al. identified 217 age-related plasma proteins using the SOMAscan proteomic assay [2]. However, these two proteomic approaches can detect only part of the plasma proteome. In addition, these studies revealed that there is considerable inter-individual variability in many plasma proteins, likely reflecting the health status of individuals. When we compared our listed proteins with those identified by the 2D-DIGE and SOMAscan approaches, we found little overlap. Recently, Gan et al. analyzed serum samples from young and old mice using MS/MS, and reported that the top 10 differentially expressed proteins were α2HS-glycoprotein, lumican (LUM), THBS4, α-fetoprotein, SPARCL1, COMP, pregnancy zone protein, complement factor H (CFH), α1β glycoprotein and complement factor 1 [14]. Surprisingly, five of these proteins overlapped with our candidate proteins: LUM, THBS4, SPARCL1, COMP and CFH. Among them, LUM, THBS4, SPARCL1 and COMP were enriched in the blood of young mice, while CFH was more abundant in the blood of old mice. Although many plasma proteins are known to be altered in aged mice and/or humans, their functions in the aging process have not yet been characterized in detail.

In order to identify novel circulating proteins that may be linked with bone loss, we tested the effects of the identified differentially expressed proteins on osteoclast differentiation. Among the seven selected candidates, CDH-13 was found to inhibit osteoclast differentiation *in vitro* and to preserve the BMD of the femur in aged mice. The cadherin family members are cell-cell adhesion molecules that have cadherin repeats in their extracellular domains. The large cadherin proteins are key contributors to morphogenetic processes during development and tissue organization [34]. Several cadherins are cleaved by proteases, and their cleaved fragments are involved in various biological processes. For example, the matrix metalloproteinase-induced cleavage of E-cadherin is required for apical cell extrusion and cancer progression [35–38]. The extracellular fragment of cadherin-11, which is cleaved by A Disintegrin And Metalloprotease 13 (ADAM13), promotes cell migration [39]. CDH-13 has been demonstrated to circulate in plasma, which may indicate that it is proteolytically cleaved and released from a yet-unknown source to enter the bloodstream.

CDH-13 has been reported to suppress cell migration, neuronal outgrowth and axon guidance through homophilic and heterophilic interactions [20, 40–42]. It also serves as a receptor for low-density lipoprotein, functions as a receptor for adiponectin to support angiogenesis and protect from stress-induced pathological cardiac remodeling [43–46], and binds to insulin granules to contribute to insulin secretion [47]. Our results demonstrated that CDH-13 inhibits osteoclast differentiation by blocking RANKL signaling (Figure 4). Although the underlying molecular mechanisms remain to be elucidated, we speculate that plasma CDH-13 may function as a decoy receptor of RANKL or as a RANK receptor antagonist.

We also found that CDH-13 was enriched in the blood of young mice and helped to preserve bone mass by inhibiting RANKL-induced osteoclast differentiation. Multiple circulating factors regulate bone mass [48]. Our results suggested that CDH-13 is an age-related bone factor, and that lower levels of CDH-13 disrupt the balance of bone remodeling and promote age-related bone loss. Since the inhibition of RANKL has long been recognized as a therapeutic strategy for osteoporosis, our findings suggest that CDH-13 could be used as a novel therapeutic molecule to inhibit bone loss.

## MATERIALS AND METHODS

### Reagents

All the chemicals used in this study were of sequencing grade and were purchased from Sigma (St. Louis, MO, USA) unless otherwise specified.

### Mouse plasma sample preparation

Plasma samples from healthy young (2-month-old) and aged (21- to 23-month-old) C57BL/6J mice were obtained from the Biomedical Mouse Resource Center at the Korea Research Institute of Bioscience and Biotechnology (KRIBB) and stored at –80 °C until further use. Plasma samples from every three mice were combined to generate pooled plasma sample sets. Six mouse plasma sets were collected from each group (young and old).

### Immunoaffinity depletion of high-abundance proteins

The three most abundant mouse plasma proteins (albumin, IgG and transferrin) were depleted with an Agilent Multiple Affinity Removal System Column on an Agilent LC system (1290 Infinity; Agilent Technologies, Santa Clara, CA, USA). Briefly, 20 μL of plasma was diluted to 100 μL in Buffer A mixed with protease inhibitor, filtered through a 0.22-μm microcentrifuge filter, injected onto the column, and depleted with the manufacturer’s recommended protocols and buffers. The flow-through fractions containing unbound proteins were collected and stored at −20 °C until further use. The depleted mouse plasma samples were then concentrated through 3-kDa filter spin columns (Amicon® Ultra-0.5 filter) and stored at −80 °C until further use.

### In-solution trypsin digestion

In-solution trypsin digestion was performed as described in our recent publication with little modification [49]. Briefly, the protein concentration was determined using the Bradford method, and 300 μg of protein from young or old mouse plasma was resuspended in 8 M urea, 75 mM NaCl, 50 mM Tris-HCl (pH 7.5) and a protease inhibitor mix. The protein mixture was then reduced in tris(2-carboxyethyl) phosphine (Thermo Fisher Scientific) at a final concentration of 5 mM for 60 min at room temperature (25 °C). Cysteines were alkylated with iodoacetamide at a final concentration of 15 mM for 60 min at room temperature in the dark. The sample was diluted 10-fold with 50 mM Tris-HCl to reduce the concentration of urea to 0.8 M or less, and then was digested overnight with trypsin (1:50 enzyme:substrate ratio) at 37 °C. The digested sample was allowed to cool at room temperature, and digestion was quenched by acidification with 0.5% trifluoroacetic acid. The sample was purified/desalted with a C18 MacroSpin column (The Nest Group Inc., Southborough, MA, USA), divided into two equal parts (one for OFFGEL separation and the other for high-pH fractionation; ~150 μg of peptides per portion), dried in vacuo and stored at –20 °C for further use.

### OFFGEL separation

For pI-based peptide separation, a 3100 OFFGEL Fractionator and an OFFGEL Kit pH 3-10 (both from Agilent Technologies) with a 12-well setup were used according to the manufacturer’s instructions. First, 12-cm-long immobilized pH-gradient gel strips with a non-linear pH gradient ranging from 3 to 10 were rehydrated in the assembled device with 40 μL of focusing buffer per well for 15 min prior to sample loading. Then, 150 μg of the tryptic digest from young or aged mouse plasma was diluted in focusing buffer to a final volume of 1.8 mL, and 150 μL of each peptide sample was loaded into each well. The sample was electrofocused with a maximum current of 50 μA and a maximum power of 200 mW until 50 kVh was reached after 24 h. The recovered fractions (volumes between 100 and 200 μL) were acidified with trifluoroacetic acid. The OFFGEL fractions were subsequently purified/desalted on a Pierce^®^ C18 Spin column (Thermo Scientific, Rockford, IL, USA), dried in vacuo and stored at –20 °C for MS analysis.

### High-pH reversed-phase fractionation

Tryptic digests of plasma samples were fractionated with a high-pH RPLC system (Agilent Technologies, 1290 Infinity LC System). The fractionations were performed with an XBridge C18 column (4.6 mm internal diameter × 250 mm length; pore size 300 Å and particle size 5 μm; Waters Corporation, Milford, MA, USA) at a flow rate of 0.5 mL/min. The phases consisted of 10 mM ammonium formate (pH 10) as mobile phase A and 10 mM ammonium formate in 90% acetonitrile (pH 10) as mobile phase B. Individual plasma digests were dissolved in 20 μL of mobile phase A and then injected through an autosampler into a 20-μL sample loop. The following program was used: hold at 5% mobile phase B for 10 min; apply gradients of 5% to 40% B for 38.5 min and 40% to 70% B for 14 min; wash the column with a hold at 70% B for 10 min; and finally re-equilibrate the column with a gradient of 70% to 5% B for 20 min. During fractionation, the peptide elution profile was monitored based on the ultraviolet absorbance at 215 nm, and the eluents were collected every 0.4 min into separate 1.5-mL tubes, for a total of 168 initial fractions. We pooled these 168 fractions into 12 concatenated fractions by combining each set of an arithmetic sequence with a common difference of 12 into one concatenated fraction. The final 12 major fractions were dried in a SpeedVac and stored at −20 °C for further use.

### MS analysis

Peptide samples were reconstituted with 0.4% acetic acid and injected from a cooled (10 °C) autosampler into a reversed-phase Magic C18aq column (15 cm × 75 μm, 5 μm, 200 Å, packed in-house; Michrom BioResources, Auburn, CA, USA) on an Eksigent NanoLC-Ultra 1D Plus system at a flow rate of 300 nL/min. Prior to use, the column was equilibrated with 95% buffer A (0.1% formic acid in water) and 5% buffer B (0.1% formic acid in acetonitrile). The peptides were eluted with a linear gradient from 5% to 40% buffer B over 120 min, followed by an organic wash and aqueous re-equilibration at a flow rate of 300 nL/min, with a total run time of 170 min. The LC system was coupled to a Q Exactive quadrupole mass spectrometer (Thermo Fisher Scientific, Bremen, Germany) operated in data-dependent acquisition mode. Survey full-scan MS spectra (m/z 300-2000) were acquired with a resolution of 70,000. The utilized source ionization parameters were as follows: spray voltage, 1.9 kV; capillary temperature, 275 °C; and s-lens level, 44.0. The MS peak width at half height was < 30 s. The MS/MS spectra of the 12 most intense ions from the MS1 scan with a charge state ≥ 2 were acquired with the following options: resolution, 17,500; isolation width, 2.0 m/z; normalized collision energy, 27%; ion selection threshold, 4.00E+03 counts; and peptide match, ‘prefer’. A previous report indicated that the quantification of proteins was independent of the dynamic exclusion settings of the data-dependent acquisition mode, and that extension of the dynamic exclusion duration to 90 s allowed greater quantification of less-abundant proteins [50]. Thus, in our MS configuration, the dynamic exclusion duration was set to 90 s. The volumes of the injected fractions were adjusted so that approximately 1 μg of tryptic peptides would be consistently injected. The peptide concentrations were estimated based on the assumption of quantitative recovery and an equal distribution of peptides among all fractions.

### Database searches and data processing

The RAW files obtained from the Q Exactive mass spectrometer were converted into mgf files by means of ProteoWizard 3.0 (MSConvert). To identify peptides and proteins, we used the MSGF^+^ (v9881) search engine to compare the mgf files with the UniprotKB (SwissProt only) mouse database (July 2014). The search engine settings were as follows: semitrypsin; 15 ppm as MS1; 3 as instrument method; 0.2 as isotopeErrorRange; allow to decoy database search; variable modification, oxidation of methionine (+15.9949 Da); and fixed modification, carbamidomethyl of cysteine (+57.0215 Da). The false discovery rate was set to 1% at the peptide-spectrum match level. The search output files were filtered and assembled, and parsimonious protein identifications were determined with IDPicker 3.1. The false discovery rate of each spectrum, peptide and protein was set to ≤ 1%, with a minimum of one unique peptide and spectrum.

### Detection of differentially expressed plasma proteins through spectral counting

To determine the differential plasma protein abundances between young and old mice, we used spectral count statistics as applied with the G-test [33]. The spectral count of each protein was corrected with the Yates correction for continuity. Proteins with G-values larger than 3.841 were regarded as differentially expressed at P < 0.05, according to the χ2-distribution. SAM analysis, which is analogous to the t-test but includes a resampling-based permutation procedure, was performed in R (r-project.org; version 3.4.2) package “samr” (version 2.0) with the false discovery rate set to 2.0% [51].

### Enzyme-linked immunosorbent assays

Commercial enzyme-linked immunosorbent assay kits were used to measure the levels of the following candidate proteins in blood: ANTXR2 (MBS2533474, Mybiosource), CDH-13 (MBS2883514, Mybiosource), COMP (MBS2886878, Mybiosource), CD163 (MBS2885730, Mybiosource), DKK3 (DY1118, R&D Systems), periostin (MBS824594, Mybiosource) and secretogranin-1 (MBS913309, Mybiosource).

### Production and purification of CDH-13

Recombinant ANTXR2, CDH-13, COMP, CD163, DKK3, periostin and secretogranin-1 were purchased from Y-Biologics (Daejeon, Korea). The appropriate DNA fragments were cloned into the N293F-FC vector, and proteins were generated in HEK293F cells.

### Osteoclast differentiation

BMMs from murine bone marrow precursors derived from six- to eight-week-old male C57BL/6 mice (The Jackson Laboratory) were prepared as previously described [52]. BMMs were treated with M-CSF (30 ng/mL) and RANKL (100 ng/mL) for three to four days, and matured osteoclasts were fixed and stained for the presence of tartrate-resistant acid phosphatase (TRAP) with a TRAP staining kit (Sigma-Aldrich). Osteoclasts were defined as pink TRAP-positive multinucleated cells (i.e., having more than five nuclei). The results of the osteoclast formation assays are presented as the mean of three independent experiments done in triplicate ± the standard deviation (SD) of the mean.

### Resorption assay

Mature osteoclasts were seeded on dentin slices and cultured with 30 ng/mL M-CSF and 100 ng/mL RANKL for two days. The dentin slices were then mechanically agitated to remove the cells. Subsequently, the slices were stained with a hematoxylin solution and Gill No. 3 for 10 min, and were washed with water. The resorbed pit area was quantified with Image J software (National Institutes of Health, Bethesda, MD, USA). Four bone slices were measured under each experimental condition.

### Western blot analysis

Cells were vortexed five times during a 30-min period on ice in a lysis buffer containing 20 mM 4-(2-hydroxyethyl)-1-piperazineethanesulfonic acid (HEPES, pH 7.0), 150 mM NaCl, 1% Triton X-100, 10% glycerol, proteinase inhibitors (1 mM phenylmethanesulfonyl fluoride and 1 μg/mL leupeptin and aprotinin) and phosphatase inhibitors (1 mM NaVO_4_ and 1 mM NaF). The samples were centrifuged at 14,000 rpm for 20 min, and the supernatants were boiled in 6X sodium dodecyl sulfate sample buffer containing 0.6 M dithiothreitol. The cell lysates were separated with 10% sodium dodecyl sulfate polyacrylamide gel electrophoresis and electrotransferred to a polyvinylidene difluoride membrane (Millipore, Billerica, MA, USA). The membranes were blocked with 5% bovine serum albumin in Tris-buffered saline containing 0.1% Tween-20, and were immunoblotted with primary antibodies against ERK, phospho-ERK, p38, phospho-p38, JNK, phospho-JNK, p65, phospho-p65, IκBα, PLCγ2, phospho-PLCγ2 (1:1000) and glyceraldehyde-3-phosphate dehydrogenase (1:5000) (Cell Signaling Technology, Beverly, MA, USA). The bound antibodies were reacted with horseradish peroxidase-conjugated secondary antibodies (1:5000), and the protein bands were detected with an enhanced chemiluminescence detection kit (Bio-Rad Laboratories, Hercules, CA, USA).

### Micro-CT imaging and data analysis

CT imaging was performed with a Quantum GX Micro-CT imaging system (PerkinElmer, Hopkinton, MA, USA) located at the Korea Basic Science Institute (Gwangju, Korea). The X-ray source was set to 90 kV and 88 mA, with a field of view of 10 mm (voxel size, 20 μm; scanning time, 14 min). The 3D images were integrated using the 3D Viewer software within the Quantum GX system, and the resolution was set to 4.5 μm. Images were obtained for visualization and display. Following scanning, the structural parameters of trabecular bone were evaluated with Analyze 12.0 software (AnalyzeDirect, Overland Park, KS, USA). The BMD of the femur was estimated based on a hydroxyapatite phantom (QRM-MicroCT-HA, Quality Assurance in Radiology and Medicine GmbH, Germany), which was scanned with the same parameters. The Region of Interest tool in the software was used to calculate the BMD, total volume, bone volume, bone surface area, bone surface density (bone surface area over total volume), trabecular thickness, trabecular separation and trabecular number of the femur. The values of the parameters are shown as the mean ± SD.

### Statistical analysis

Statistical analyses were performed with the Statistical Package for the Social Sciences (SPSS version 17.0). Statistical significance was calculated with Student’s unpaired t-test. Unless otherwise specified, all data are presented as the mean ± standard error of the mean (SEM), and significance is indicated as *P < 0.05, **P < 0.01 and ***P < 0.001.

## Supplementary Material

Supplementary Methods

Supplementary Figures

Supplementary Table 1
