# Supplementary material for: Plasma proteomic profiling of young and old mice reveals cadherin-13 prevents age-related bone loss
Source: Aging (Albany NY). 2020 May 12;12(9):8652–68. doi: 10.18632/aging.103184 (PMC7244053; doi:10.18632/aging.103184)
Supplement: Supplementary Methods [file aging-12-103184-s001..pdf]

SUPPLEMENTARY METHODS

Cytotoxicity assay

The toxicity of CDH-13 was determined with an MTT assay. Cells were seeded in 96-well plates, treated with 5 mg/mL MTT and incubated for 3 h at 37 °C. The medium was removed and 150 mL of MTT solvent was added (isopropanol containing 4 mL HCL and 0.1% Nonidet P-40). The plate was incubated for 15 min and read at 590 nm with a microplate reader.

Mouse calvarial preosteoblast isolation and osteoblast differentiation

Primary mouse osteoblasts were obtained from calvarial bones excised from two-day-old neonatal mice [1]. For osteoblast differentiation, the cells were cultured with osteogenic medium containing 10 mM β-glycerol phosphate (Sigma), 50 μg/mL ascorbate-2-phosphate (Sigma), 10^{-7} M dexamethasone (Sigma) and 25 ng/mL human recombinant bone morphogenetic protein 2 (R&D Systems) for 7 or 21 days.

Alkaline phosphatase (ALP) assay and Alizarine red S (ARS) staining

After 7 days of osteoblast differentiation, ALP activity was detected with 5-bromo-4-chloro-3-indolyl-phosphate/nitro blue tetrazolium (BCIP/NBT) color development substrate (Promega, Wisconsin, USA) according to the manufacturer's instructions. For the detection of mineralization, preosteoblasts were cultured in osteogenic medium for 14-21 days and stained with ARS. Briefly, cells were fixed with 4% paraformaldehyde for 10 min and stained with 2% ARS, pH 4 (Alphachem, Middlesex, UK) for 10 min. For quantification of mineralization, ARS was removed by treatment with 10% acetic acid for 30 min, heating at 85 °C for 10 min, centrifugation at 20,000 x *g* for 15 min and neutralization with 10% ammonium hydroxide. The supernatants were read at 405 nm with a microplate reader.

REFERENCE

1. Rosen CJ, Dimai HP, Vereault D, Donahue LR, Beamer WG, Farley J, Linkhart S, Linkhart T, Mohan S, Baylink DJ. Circulating and skeletal insulin-like growth factor-I (IGF-I) concentrations in two inbred strains of mice with different bone mineral densities. Bone. 1997; 21:217–23. https://doi.org/10.1016/S8756-3282(97)00143-9 PMID:9276086
