# Supplementary material for: Plasma proteomic profiling of young and old mice reveals cadherin-13 prevents age-related bone loss
Source: Aging (Albany NY). 2020 May 12;12(9):8652–68. doi: 10.18632/aging.103184 (PMC7244053; doi:10.18632/aging.103184)
Supplement: Supplementary Figures [file aging-12-103184-s002..pdf]

SUPPLEMENTARY FIGURES

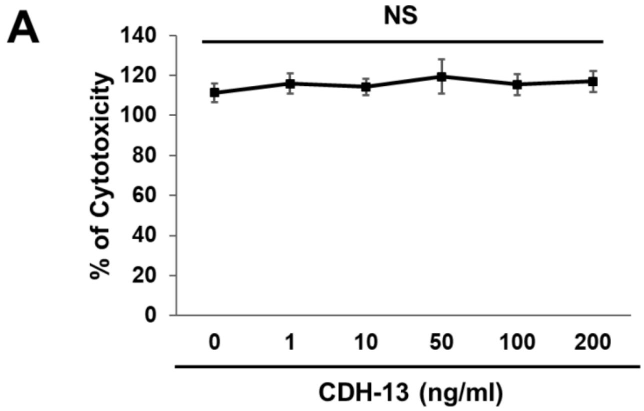

Supplementary Figure 1. (**A**) BMMs were cultured for two days in the presence of M-CSF (30 ng/mL) with CDH-13 (0, 1, 10, 100 or 200 ng/mL). The cytotoxicity of CDH-13 to BMMs was measured with a 3-(4,5-dimethylthiazol-2-yl)-2,5-diphenyltetrazolium bromide (MTT) assay. Error bars represent ± SEM. NS, not significant.

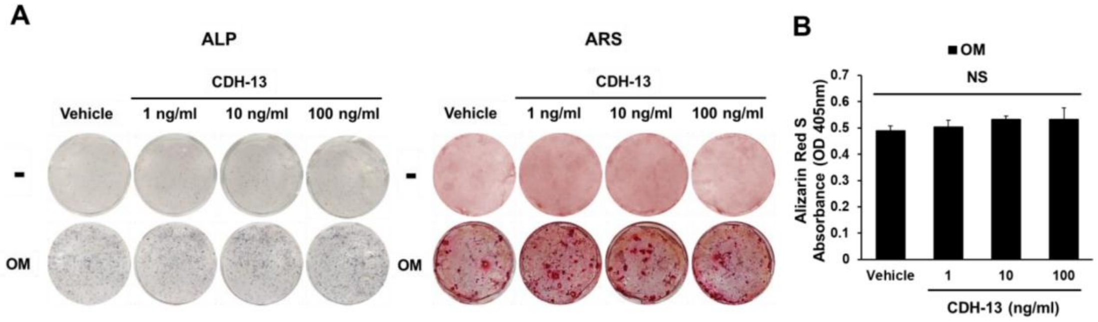

Supplementary Figure 2. CDH-13 does not alter osteoblast differentiation. (**A**) Calvarial preosteoblasts were cultured for 7 days (alkaline phosphatase [ALP] staining) or 21 days (Alizarin Red S [ARS] staining) in the presence of osteogenic medium (OM) with the vehicle or CDH-13 (1, 10 or 100 ng/mL). The osteoblasts were then stained with ALP or ARS. (**B**) The ARS content was quantified. Error bars represent the SEM. NS, not significant.
